# Case of Habitual Cough, Cured by a Severe Burn

**Published:** 1822-08

**Authors:** Thomas Ogden

**Affiliations:** Surgeon.


### Art. II.—*Case of Habitual Cough, cured by a severe Burn.* By Thomas Ogden, Surgeon.

J. Oulton, a boy, æt. six, had been troubled with cough, accompanied by profuse expectoration, from the earliest period of his life: this was always attended with such a ruttling noise, that his breathing, when asleep, might be heard in the adjoining room. He was also, about two years ago, attacked by epileptic fits, returning at intervals of nine or ten weeks; but these were always brought on, or preceded by, a deranged state of the alimentary canal, for which he occasionally took mercurial purgatives; and, during the fits, glysters always brought away very large quantities of clayey stools.*

He was in this state, when, on the 22nd of October, 1821, his clothes caught fire, and he was burnt in a most shocking manner, the skin being removed with his clothes from the whole of the thorax in front, and from a considerable portion of the abdomen. Indeed, there was so large a portion injured, that I expected him to sink under the irritative fever; but, happily, I was deceived.

* I apply the word *clayey* to denote both colour and consistence.

I dressed him with terebinthinates for the first few days, but was under the necessity of discontinuing them sooner than I intended, from their producing universal erythema of the skin. His breathing continued the same as before, until suppuration was fully established, when all at once his cough, wheezing, and expectoration, ceased; nor have any of these symptoms returned to this period, and he now enjoys a better state of health than he ever experienced previously; and he is, in fact, a fine healthy child, for, since the 20th of November last, he has had no return of his epileptic fits; nor has the state of his bowels required any medicine for a considerable time past.

Cicatrization in this case, as might be expected from the extent of skin destroyed, proceeded but slowly; nor did I feel anxious for its rapid progress, especially towards the latter part of the time, fearing a return of the cough, &c. on the stopping of this drain.

The whole period occupied by the process of cicatrization was near seven months, it proceeding regularly, but slowly, from circumference to centre; there not being left the least portion of undestroyed skin, in the midst of this extended sore, that could serve as a nucleus from whence cicatrization could extend towards the circumference.

Does not this case point out the propriety of employing perpetual blisters and moxa, in obstinate pectoral diseases, over a much larger surface, and of continuing their use for a much longer period, than has been customary?

If, during the ensuing winter, he should have any relapse of his pectoral complaints, I will again write to you.

*Ashton-under-Lyne; June 5th,* 1822.

